# Genetic and phenotypic heterogeneity in sporadic and familial forms of paroxysmal dyskinesia

**DOI:** 10.1007/s00415-012-6592-5

**Published:** 2012-06-30

**Authors:** Alexander J. A. Groffen, Thom Klapwijk, Anne-Fleur van Rootselaar, Justus L. Groen, Marina A. J. Tijssen

**Affiliations:** 1 Departments of Clinical Genetics and Functional Genomics, Center of Neurogenomics and Cognitive Research (CNCR), VU University and VU Medical Center, Amsterdam, The Netherlands; 2 Department of Neurology, Academic Medical Center, University of Amsterdam, Amsterdam, The Netherlands; 3 Department of Neurology AB 51, University Medical Center Groningen, P.O. Box 30.001, 9700 RB Groningen, The Netherlands

**Keywords:** Paroxysmal kinesigenic dyskinesia, Infantile convulsions and paroxysmal choreoathetosis, Benign familial neonatal convulsions, Migraine

## Abstract

Paroxysmal dyskinesia (PxD) is a group of movement disorders characterized by recurrent episodes of involuntary movements. Familial paroxysmal kinesigenic dyskinesia (PKD) is caused by *PRRT2* mutations, but a distinct etiology has been suggested for sporadic PKD. Here we describe a cohort of patients collected from our movement disorders outpatient clinic in the period 1996–2011. Fifteen patients with sporadic PxD and 23 subjects from three pedigrees with familial PKD were screened for mutations in candidate genes. *PRRT2* mutations co-segregated with PKD in two families and occurred in two sporadic cases of PKD. No mutations were detected in patients with non-kinesigenic or exertion-induced dyskinesia, and none in other candidate genes including *PNKD1* (*MR*-*1*) and *SLC2A1* (*GLUT1)*. Thus, *PRRT2* mutations also cause sporadic PKD as might be expected given the variable expressivity and reduced penetrance observed in familial PKD. Further genetic heterogeneity is suggested by the absence of candidate gene mutations in both sporadic and familial PKD suggesting a contribution of other genes or non-coding regions.

**Electronic supplementary material:**

The online version of this article (doi:10.1007/s00415-012-6592-5) contains supplementary material, which is available to authorized users.

## Introduction

Paroxysmal dyskinesias (PxDs) are characterized by recurrent episodes of abnormal involuntary movements [1]. The movement disorder may clinically present as dystonia, chorea, ballism or a mixture of these [2]. Attacks often begin in childhood [3]. A classification scheme distinguishes four subtypes: paroxysmal kinesigenic (PKD), non-kinesigenic (PNKD), exertion-induced (PED) and hypnogenic dyskinesia (PND) [2].

In PKD, the most frequent form, attacks are precipitated by voluntary movements and usually last less than 5 min [4]. Familial PKD inherits in an autosomal dominant mode with reduced penetrance [3]. Infantile convulsions (IC) were frequently observed in the same pedigree and PKD and ICCA (IC with choreoathetosis) are now considered the same disorder [5–9]. Recently, the *PRRT2* gene was shown to cause familial PKD [10–16]. A quarter of PKD cases are sporadic. Several observations suggested that sporadic and familial forms may be etiologically distinct, featuring a higher number of attacks per day and an increased co-occurrence of migraine in sporadic patients (full reference list in Online Resource 1).

In PNKD attacks are triggered by alcohol, caffeine, fatigue or excitement [2]. Attacks typically last minutes to 4 h. Mutations in the *MR*-*1* gene have been detected in several families [17, 18]. Clonazepam or other benzodiazepines can be helpful in some *MR*-*1* mutation carrier patients [19].

PED is triggered by prolonged physical exercise [20]. The symptom duration varies from 5–30 min [1, 3]. Few families have been described [21–24] as well as some sporadic patients [25, 26]. In some families epilepsy or migraine co-occur. In nine families with PED and epilepsy, mutations were found in the *SLC2A1* gene encoding the glucose transporter GLUT1 [27, 28].

Here we investigated a cohort of 33 Dutch PxD patients and found *PRRT2* frameshift mutations in both familial and sporadic cases of PKD. Furthermore, the majority of sporadic PxD cases as well as one PKD family did not carry mutations in *PRRT2*, *MR*-*1* or *GLUT1*, suggesting a contribution of additional genes or non-coding mutations.

## Subjects and methods

This study was performed in accordance with the ethical standards laid down in the 1964 declaration of Helsinki and was approved by the Institutional Review Board of the VU Medical Center (#2009/174). Informed written consent was obtained from all participating subjects. All patients with PxD in the Academic Medical Center (AMC), diagnosed between 1996 and 2010 were asked to participate . All participants completed a standardised questionnaire including age at onset, frequency, duration, triggers, symptoms during attacks, precipitating and alleviating factors, symptoms after an attack and family and medical history. Medical records EEG (electro-encephalogram, if achieved) and imaging reports were reviewed. In PKD families some unaffected family members were also examined. For genetic analysis, we collected venous blood samples (adults) or sputum (children). Genomic DNA was isolated using the PureGene Blood core kit (Qiagen). For familial cases, co-seggregation of the phenotype with chromosome 16 was confirmed using 23 microsatellite repeats (see Online Resource 2). PCR amplification of target genes was performed using primers specified in Online Resource 3. Products were sequenced using BigDyeTerminator v3.1 reagents and analyzed in a ABI Prism 3137 genetic analyzer. All patients were tested for *PRRT2*. Patients with PKD or PED were also tested for mutations in *GLUT1*. PNKD patients were also tested for *MR*-*1* gene mutations.

## Results

### Patients

Thirty-three PxD patients were detected and 23 volunteered to participate. Sixteen patients met the criteria for PKD including seven patients with a positive family history (Table 1). The average age at onset was 14.1 years (ranging from 0 to 51) and did not differ significantly between familial and sporadic cases (12.4 and 15.4 years, respectively). There is a male predominance (63 %). Dystonia was observed in 88 % of the patients, whereas 31 % exhibited chorea. Combined dystonia and chorea was observed in 25 % of the patients. Seven patients tried carbamazepine and all experienced good control of complaints. Accompanying ailments were IC, epilepsy, migraine and tremor. It should be noted that IC were only present in the familial group.

**Table 1:** Clinical characteristics for familial/sporadic PxD cases studied and observed *PRRT2* mutations

Patient	PxD	Sex	AaO (years)	Inducer	Dystonia/chorea	Duration	Frequency	Localisation	EEG	Migraine	Medication	*PRRT2* mutation	Other genes tested
Fam. 1, II-2	PKD	F	14	Movement change	D+C	7–30 s	Daily	Left sided	N/A	–	None	c.649dupC p.R217PfsX7	
Fam. 1, II-3	PKD	M	15	Movement change	D+C	10 s	10× per day	Left sided	N/A	–	CBZ: good effect	c.649dupC p.R217PfsX7	*GLUT1*
Fam. 2, IV-2	PKD	M	8	Sudden movement	D	5–15 s	1–2× per day	Left sided	N/A	–	CBZ: good effect	None	
Fam. 2, III-1	PKD	F	10	Sudden movement	D	10–15 s	Weekly	Right sided	N/A	–	DPhT: moderate effect	None	
Fam. 2, II-4	PKD	M	10	Sudden movement	D	10 s	N/A	Right arm	N/A	–	None	None	
Fam. 3, III-1	PKD	M	15	Sudden movement	D	10 s	Daily	Left leg	Epilepsy	–	CBZ: good effect	c.649C>T p.R217X	*GLUT1*
Fam. 3, II-4	PKD	M	15	Sudden movement	D	10–15 s	3× per week	Left sided	Normal	–	None	c.649C>T p.R217X	
1, Sporadic	PKD	F	3	Playing	D	Minutes	Daily	Right sided	Normal	–	None	No DNA	
2, Sporadic	PKD	F	0	Movement change	D+C	1.5 min	Several per day	Generalized	Normal	–	None	c.3698T>C in 3′UTR	*GLUT1*
3, Sporadic	PKD	F	51	Walking	C	1 s	2–10× per day	Both legs	Normal	–	CBZ: good effect	None	*GLUT1*, *MR*-*1*
4, Sporadic	PKD	M	13	Sudden movement	D	10 s	1–2× per day	Right sided	N/A	–	CBZ: good effect	c.649delC p.R217PfsX12	
5, Sporadic	PKD	M	14	Start of movement	D+C	15 s	10× per day	Alternating sides	N/A	–	CBZ: good effect	None	*GLUT1*, *MR*-*1*
6, Sporadic	PKD	M	11	Movement change	D	10 s –1 min	1–2× per day	Generalized	N/A	–	CBZ: good effect	c.649dupC p.R217PfsX7	
7, Sporadic	PKD	F	29	Small changes of movement	C	Seconds–minutes	Several per day	Left arm	Non-epileptiform PT abnormalities	Yes	TBZ: moderate effect	None	*GLUT1*, *MR*-*1*
8, Sporadic	PKD	M	12	Sudden movement	D	10–20 s	5× per day	Right sided	N/A	–	None	None	*GLUT1*, *MR*-*1*
9, Sporadic	PKD	M	6	N/A	D	<1 min	Daily	Generalized	Normal	Yes	DPK: good effect	No DNA	
10, Sporadic	PNKD	F	0	No clear inducers	D+C	Minutes	N/A	Generalized	Normal	Yes	CBZ, BDZ, DPK, DPhT: moderate effect	None	*MR*-*1*
11, Sporadic	PNKD	F	0	Physical exercise, cold	D	Several hours	Daily–weekly	Generalized	Suspect for epilepsy	–	BDZs, unknown effect	None	*MR*-*1*
12, Sporadic	PNKD	F	9	Fatigue	D	Minutes to hours	Several a month	Right sided	N/A	Yes	DPK, lot of side effects	None	*MR*-*1*
13, Sporadic	PNKD	M	10	No clear inducers	D	30 min	Daily	Right sided	Normal	–	Anti-PD medication, CBZ, BDZ: no effect	None	*MR*-*1*
14, Sporadic	PED	F	16	Prolonged exercise	D	Up to 3 h	7× per quarter	Right arm	PED-like	–	Clobazam	None	*GLUT1*, *MR*-*1*

*AaO* age at onset, *PT* pariotemporal, *CBZ* carbamazepine, *TBZ* tetrabenazide, *DPhT* diphantoine, *DPK* depakine, *PD* Parkinsons disease

**Fig. 1:**
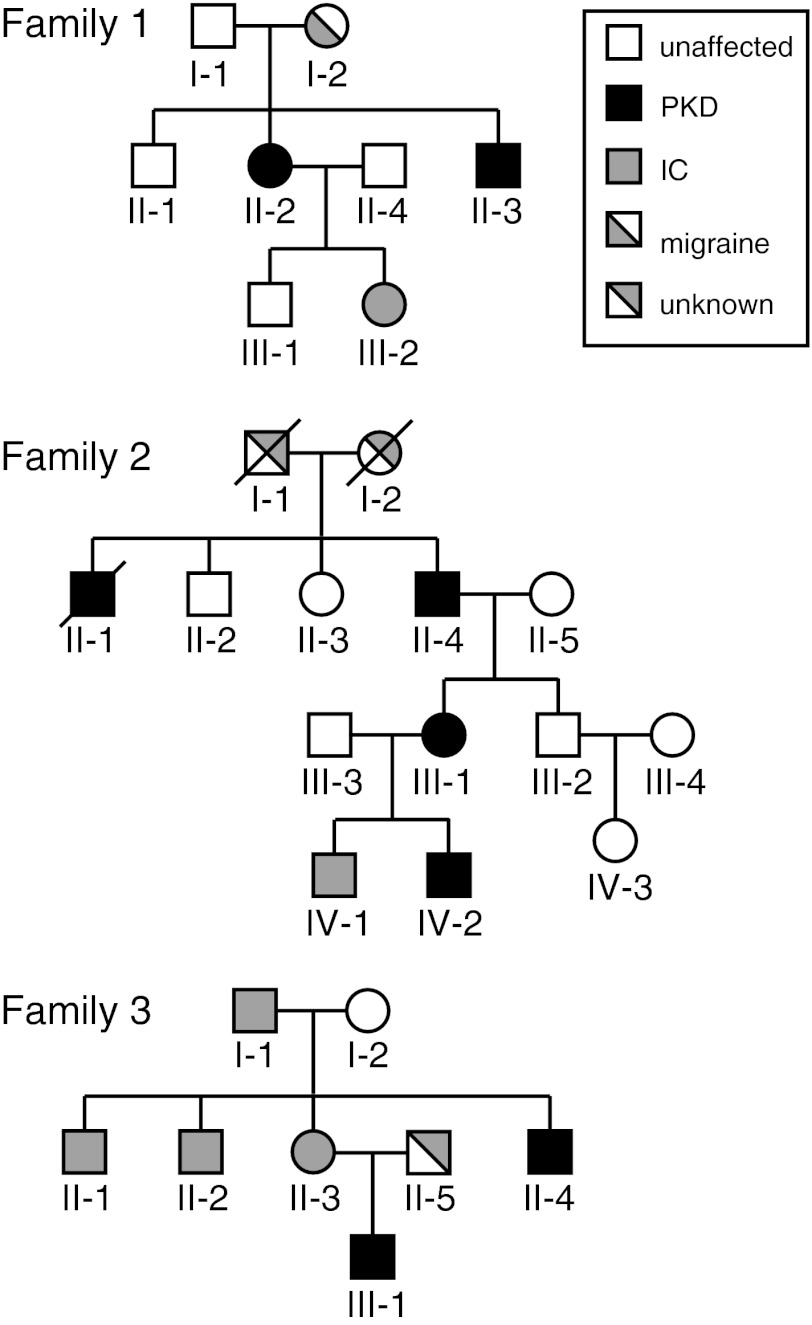
Pedigrees with familial PKD. Convulsions co-occurred in all families

The first family of Indonesian descent (Fig. 1) contains two patients with PKD, one with IC and one with migraine. The index patient (II-2), a 37-year-old woman, reported attacks of 10 s or less since the age of 12 years. Attacks consisted of a right-sided dystonic posture of her neck, jaw, hand, arm and leg. She experienced a preceding sensation and could abort attacks, the frequency of which decreased without medication. Her younger brother (III-3) had an identical phenotype. His attacks stopped with carbamazepine (50 mg per 2 days). Her daughter (IV-2) had IC without EEG abnormalities at the age of 6 months. In the past her mother (I-2) had migraine.

The second family consists of three PKD patients of Dutch origin. The index patient (IV-2), a 13-year-old boy had paroxysms since the age of 10 years after switching from rest to action. The attacks consisted of dystonic posture of his left arm followed by his mouth and leg. He had tremors of the hands and sudden involuntary movement of his neck. The patient experienced a preceding sensation and could abort an attack. The attacks presented less than five times a day, lasted 10–15 s and ceased with carbamazepine (50 mg dd). His mother (III-1) experienced similar attacks in her youth (10–20 years). She also reported a febrile convulsion at 1 year of age. The grandfather (II-4) had similar attacks when he was young and also exhibited tremors of the hands. A great-uncle (II-1) also experienced involuntary movements, but specifics were not possible to collect. The older brother (IV-1) had a febrile convulsion at 1 year of age.

The third family includes two PKD patients. The index patient (III-1) had PKD since age 15 years. The attack consisted of left-sided slow kicking movements causing him to fall. Occasionally a sensory warning preceded the attack, but he was unable to prevent attacks. They stopped with carbamazepine (2 dd 200 mg). He was previously diagnosed with benign familial infantile convulsions (BFIC) treated with valproic acid, and had a single generalized tonic–clonic seizure at age 13 years and twice again at age 14 years. Interictal EEG showed series of epileptic discharges over the right frontocentral area, especially during drowsiness and hyperventilation [29]. Considering the PKD and seizures, the diagnosis ICCA was made.

Besides PKD patients, our cohort contained four sporadic PNKD patients and one female PED patient. The average onset age of PNKD was 4.75 (0–10) years similar to previous findings [1, 26, 30]. Again, dystonia was the most common expression. Two patients tried carbamazepine without effect. Two patients had migraine. The female PED patient had isolated dystonia. Complaints started at age 16 years; attacks gradually decreased and she had her last attack at age 23 years. During an attack she took clobazam to stop the complaints. It is common to have no family history for PED [26].

Several patients underwent a brain CT-scan or MRI (Table 1). All were normal. Only in the PED patient three small lacunar lesions appeared in the right sided basal ganglia, especially the global pallidus and adjacent to the internal capsule. In EEG registrations, most of the patients showed no epileptic discharges. Clinical epilepsy was confirmed by neurophysiological investigation in only one PKD patient (III-1 of family 3) (Table 1). In a patient with PNKD and mental retardation an EEG showed indications for epilepsy, but clinically no convulsions ever occurred.

### Mutation detection

For familial PKD, *PRRT2* mutations have been observed in multiple Han Chinese families [10–13, 16, 31]. Consistently, we observed *PRRT2* mutations in family 1 (c.649dupC) and 3 (c.649C>T, introducing an opal stop codon replacing proline 217). In family 2 we did not find any mutation, although the phenotype co-seggregated with a region of chromosome 16p distal from marker D16S685 (see Online Resource 2).

**Fig. 2:**
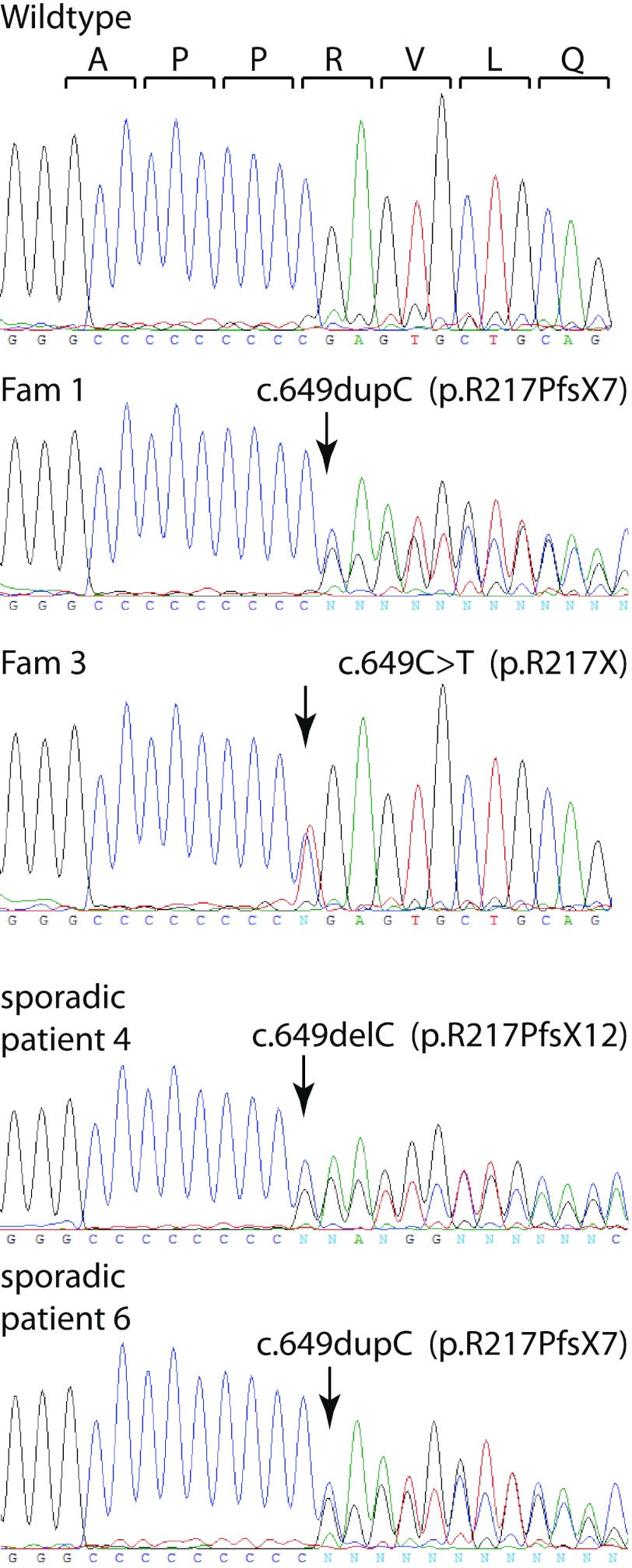
Sequence traces of observed *PRRT2* mutations. *Top trace* indicates the wildtype sequence and the corresponding open reading frame. *Arrows* indicate mutated residues

In addition, two sporadic PKD patients carried frameshift mutations in *PRRT2* (Table 1; Fig. 2) c.649delC (patient 4) and c.649dupC (patient 6). Another variation, c.3698T>C, was observed in the 3′UTR in exon 4. Although this variation is not reported in dbSNP or the 1,000 genome database it is not predicted to have major effects on *PRRT2* gene function.

Six out of nine cases of sporadic PKD were not associated with mutations in *PRRT2*. We also did not detect mutations in other candidate genes tested including *MR*-*1* (known to cause PNKD) and *GLUT1* (known to cause PED and sporadic PKD) [32].

## Discussion

We investigated the clinical and genetic aspects of 21 PxD patients seen over the last 15 years in our third opinion movement disorder out-patient clinic. This cohort comprised 16 patients with PKD, four with PNKD and one with PED. Familial PKD co-seggregated with the known locus in all pedigrees. *PRRT2* truncating mutations were identified in two families and two sporadic cases.

In our study 53 % of the PKD patients were sporadic cases, whereas only 4.8 % of published cases were sporadic [4, 33]. Possibly sporadic forms are underrepresented, because linkage and gene identification studies preferentially focussed on familial forms. Other clinical aspects of patients in our cohort were similar to those of in total 681 published PKD patients (see Online Resource 1). The mean age at onset was similar (10.4 years on average) and as in our cohort, dystonia was also the most common movement disorder in literature (seen in 43 % of patients). Both in literature (30 %) and in our findings PKD and IC are correlated, especially in the familial forms [9].

The clinical features of sporadic and familial PKD patients were similar, although IC were observed only in the familial group. Migraine occurred in two of nine sporadic PKD cases and in one PRRT2 mutation carrier in family 1 (I-2). The attacks were preceded by an aura in 93 % of sporadic patients and in all adult familial patients questioned.

Despite the previous idea that sporadic and familial PKD represent distinct diseases, we observed identical mutations in sporadic and familial PKD [11–13]. This is consistent with a recent study [16]. A pseudo-sporadic occurrence is not surprising in view of the reduced penetrance of familial PKD combined with the variable clinical expression that includes IC, BFIC and migraine [14, 15].

The lack of *PRRT2* mutations in four out of seven sporadic cases from our cohort suggests that additional PKD genes exist. In another recent study, seven out of eight sporadic cases were negative for *PRRT2* mutations [16]. Similarly, familial PKD cases occur without *PRRT2* mutations both in our study and previous investigations [16]. This suggests a contribution of other genes which may co-segregate with the same locus as *PRRT2* or alternatively of non-coding mutations that may affect *PRRT2* gene function.

## Electronic supplementary material

Below is the link to the electronic supplementary material.

Supplementary material 1 (DOC 70 kb)

Supplementary material 2 (DOC 258 kb)

Supplementary material 3 (DOC 93 kb)
