# Supplementary material for: Genetic and phenotypic heterogeneity in sporadic and familial forms of paroxysmal dyskinesia
Source: J Neurol. 2012 Jun 30;260(1):93–9. doi: 10.1007/s00415-012-6592-5 (PMC3535363; doi:10.1007/s00415-012-6592-5)
Supplement: Supplementary file 1 — Supplementary material 1 (DOC 70 kb) [file 415_2012_6592_MOESM1_ESM.doc]

**Online Resource 1:**

To compare the clinical characteristics between our cohort and 681 previously reported cases, we used data from the following references [1-23].

1. Goodenough DJ, Fariello RG, Annis BL, Chun RW (1978) Familial and acquired paroxysmal dyskinesias. A proposed classification with delineation of clinical features. Arch Neurol 35:827-831

2. Lee HY, Huang Y, Bruneau N, Roll P, Robertson EDO, Hermann M, Quinn E, Maas J, Edwards R, Ashizawa T, ..., Ptacek LJ (2012) Mutations in the gene PRRT2 cause paroxysmal kinesigenic dyskinesia with infantile convulsions. Cell Reports

3. Tan LC, Tan AK, Tjia H (1998) Paroxysmal kinesigenic choreoathetosis in Singapore and its relationship to epilepsy. Clin Neurol Neurosurg 100:187-192

4. Bruno MK, Hallett M, Gwinn-Hardy K, Sorensen B, Considine E, Tucker S, Lynch DR, Mathews KD, Swoboda KJ, Harris J, Soong BW, Ashizawa T, Jankovic J, Renner D, Fu YH, Ptacek LJ (2004) Clinical evaluation of idiopathic paroxysmal kinesigenic dyskinesia: new diagnostic criteria. Neurology 63:2280-2287

5. Demirkiran M, Jankovic J (1995) Paroxysmal dyskinesias: clinical features and classification. Ann Neurol 38:571-579

6. Hattori H, Fujii T, Nigami H, Higuchi Y, Tsuji M, Hamada Y (2000) Co-segregation of benign infantile convulsions and paroxysmal kinesigenic choreoathetosis. Brain Dev 22:432-435

7. Houser MK, Soland VL, Bhatia KP, Quinn NP, Marsden CD (1999) Paroxysmal kinesigenic choreoathetosis: a report of 26 patients. J Neurol 246:120-126

8. Lee WL, Tay A, Ong HT, Goh LM, Monaco AP, Szepetowski P (1998) Association of infantile convulsions with paroxysmal dyskinesias (ICCA syndrome): confirmation of linkage to human chromosome 16p12-q12 in a Chinese family. Hum Genet 103:608-612

9. Spacey SD, Valente EM, Wali GM, Warner TT, Jarman PR, Schapira AH, Dixon PH, Davis MB, Bhatia KP, Wood NW (2002) Genetic and clinical heterogeneity in paroxysmal kinesigenic dyskinesia: evidence for a third EKD gene. Mov Disord 17:717-725

10. Swoboda KJ, Soong B, McKenna C, Brunt ER, Litt M, Bale JF, Jr., Ashizawa T, Bennett LB, Bowcock AM, Roach ES, Gerson D, Matsuura T, Heydemann PT, Nespeca MP, Jankovic J, Leppert M, Ptacek LJ (2000) Paroxysmal kinesigenic dyskinesia and infantile convulsions: clinical and linkage studies. Neurology 55:224-230

11. Valente EM, Spacey SD, Wali GM, Bhatia KP, Dixon PH, Wood NW, Davis MB (2000) A second paroxysmal kinesigenic choreoathetosis locus (EKD2) mapping on 16q13-q22.1 indicates a family of genes which give rise to paroxysmal disorders on human chromosome 16. Brain 123 ( Pt 10):2040-2045

12. Wang X, Sun W, Zhu X, Li L, Du T, Mao W, Wu X, Wei H, Zhu S, Sun Y, Liu Y, Niu N, Wang Y (2010) Paroxysmal kinesigenic choreoathetosis: evidence of linkage to the pericentromeric region of chromosome 16 in four Chinese families. Eur J Neurol 17:800-807

13. Nagamitsu S, Matsuishi T, Hashimoto K, Yamashita Y, Aihara M, Shimizu K, Mizuguchi M, Iwamoto H, Saitoh S, Hirano Y, Kato H, Fukuyama Y, Shimada M (1999) Multicenter study of paroxysmal dyskinesias in Japan--clinical and pedigree analysis. Mov Disord 14:658-663

14. Tomita H, Nagamitsu S, Wakui K, Fukushima Y, Yamada K, Sadamatsu M, Masui A, Konishi T, Matsuishi T, Aihara M, Shimizu K, Hashimoto K, Mineta M, Matsushima M, Tsujita T, Saito M, Tanaka H, Tsuji S, Takagi T, Nakamura Y, Nanko S, Kato N, Nakane Y, Niikawa N (1999) Paroxysmal kinesigenic choreoathetosis locus maps to chromosome 16p11.2-q12.1. Am J Hum Genet 65:1688-1697

15. Zhou J, Li G, Chen C, Liu D, Xiao B (2008) Familial pure paroxysmal kinesigenic dyskinesia in Han population from the Chinese mainland: a new subtype? Epilepsy Res 80:171-179

16. Bennett LB, Roach ES, Bowcock AM (2000) A locus for paroxysmal kinesigenic dyskinesia maps to human chromosome 16. Neurology 54:125-130

17. Callenbach PM, de Coo RF, Vein AA, Arts WF, Oosterwijk J, Hageman G, ten Houten R, Terwindt GM, Lindhout D, Frants RR, Brouwer OF (2002) Benign familial infantile convulsions: a clinical study of seven Dutch families. Eur J Paediatr Neurol 6:269-283

18. Cuenca-Leon E, Cormand B, Thomson T, Macaya A (2002) Paroxysmal kinesigenic dyskinesia and generalized seizures: clinical and genetic analysis in a Spanish pedigree. Neuropediatrics 33:288-293

19. Ohmori I, Ohtsuka Y, Ogino T, Yoshinaga H, Kobayashi K, Oka E (2002) The relationship between paroxysmal kinesigenic choreoathetosis and epilepsy. Neuropediatrics 33:15-20

20. Plant G (1983) Focal paroxysmal kinesigenic choreoathetosis. J Neurol Neurosurg Psychiatry 46:345-348

21. Zorzi G, Conti C, Erba A, Granata T, Angelini L, Nardocci N (2003) Paroxysmal dyskinesias in childhood. Pediatr Neurol 28:168-172

22. Fahn S (1994) The paroxysmal dyskinesias. In: Marsden C, Fahn S (eds) Movement disorders. Butterworth-Henemann Ltd., Oxford, pp 310-345

23. Zhou X, Ma A, Liu X, Huang C, Zhang Y, Shi R, Mao S, Geng T, Li S (2006) Infantile seizures and other epileptic phenotypes in a Chinese family with a missense mutation of KCNQ2. Eur J Pediatr 165:691-695
