# Supplementary material for: Genetic and phenotypic heterogeneity in sporadic and familial forms of paroxysmal dyskinesia
Source: J Neurol. 2012 Jun 30;260(1):93–9. doi: 10.1007/s00415-012-6592-5 (PMC3535363; doi:10.1007/s00415-012-6592-5)
Supplement: Supplementary file 2 — Supplementary material 2 (DOC 258 kb) [file 415_2012_6592_MOESM2_ESM.doc]

**Online Resource 2:** Despite the absence of PRRT2 mutations, the PKD / IC phenotype co-segregated in family 2 with a region of chromosome 16p, distal from D16S685. Asterisks indicate subjects from which a DNA sample was collected. The DNA of subject IV-3 was of poor quality, leading to incomplete haplotype determination. Recombination events are indicated by horizontal lines. Dashed rectangle: chromosomal region co-segregating with the phenotypes.
