# Supplementary material for: Genetic and phenotypic heterogeneity in sporadic and familial forms of paroxysmal dyskinesia
Source: J Neurol. 2012 Jun 30;260(1):93–9. doi: 10.1007/s00415-012-6592-5 (PMC3535363; doi:10.1007/s00415-012-6592-5)
Supplement: Supplementary file 3 — Supplementary material 3 (DOC 93 kb) [file 415_2012_6592_MOESM3_ESM.doc]

**Online Resource 3:** Summary of oligonucleotide sequences used to amplify genomic DNA fragments.

|  |  |  |  |  |  | **Amplicon** |  |
| --- | --- | --- | --- | --- | --- | --- | --- |
| **Gene** | **Exon(s)** | **Fw** | **Forward primer sequence** | **Rev** | **Reverse primer sequence** | **size (bp)** | **Reference** |
| PRRT2 | exon 1 | sg505 | ttgcctgggtaacgcgtggct | sg506 | acacccgcattcccgtgcagt | 329 | [1] |
|  | exon 2.1 | sg507 | caattgggcctgcagtgctgag | sg508 | ggtttggacactgtttcttggcat | 467 | [1] |
|  | exon 2.2 | sg509 | ggaggggaatcaaaggccaactg | sg510 | tcaaccagctgctgcagcactc | 398 | [1] |
|  | exon 2.3 | sg511 | gaaaagcaagagaatggggcagtg | sg512 | gattactccagaggctctattgcag | 433 | [1] |
|  | exon 3/4 | sg513 | ttctggatgacttttccacctgat | sg516 | gtggtcccagcagagaacagtc | 1062 | this study |
| PNKD (MR-1) | MR-1L/S exon 1 | MR-FW1 | gcttctgggagatgtagtttctgg | MR-Rv1 | aggaaccaagagccctgacc | 280 | [2] |
|  | MR-1L/S exon 2 | MR-FW2 | cctcccaagcccttactgc | MR-Rv2 | cccacaactttggattcaga | 363 | [2] |
|  | MR-1S exon 3 | MR-FW3 | cagctcctctggctcagg | MR-Rv3 | tgaggacttaacagtcaatagcc | 297 | [2] |
|  | MR-1S 3' UTR | MR-Fw4 | gaccgtgtgaagcagatgaa | MR-Rv4 | ctgtgagagggagcacacat | 397 | [2] |
|  | MR-1M exon 1 | MR-Fw5 | gctcaggcgactgtggac | MR-Rv5 | ttagaccaagccccttcctaac | 574 | [2] |
|  | MR-1M exon 2 | MR-Fw6 | aggagtctaggggagctaggg | MR-Rv6 | ggctggggtatgtgaagg | 256 | [2] |
|  | MR-1M exon 3 | MR-Fw 7 | gctccccttcacataccc | MR-Rv7 | tctagcaaggcgaaactgc | 254 | [2] |
|  | MR-1M exon 4 | MR-Fw8 | tcaagtgcctcttgcatcct | MR-Rv8 | ggcccgtactgacttcttga | 220 | [2] |
|  | MR-1M exon 5 | MR-Fw9 | aggtacagttgcccctcgaa | MR-Rv9 | gcaaagatccacctgctgat | 245 | [2] |
|  | MR-1M exon 6 | MR-Fw10 | atg gaagcccactctcttgt | MR-Rv10 | cggcaacatctgtgctaaaa | 387 | [2] |
|  | MR-1M exon 7 | MR-Fw11 | tgtgttatcctggcaccttg | MR-Rv11 | cctgctttctaggggagtga | 225 | [2] |
|  | MR-1M exon 8 | MR-Fw12 | ggcagggaagactgttctga | MR-Rv12 | ctgagtgcctgcccacac | 235 | [2] |
|  | MR-1M exon 9 | MR-Fw13 | tctgtcttgggtccatttcc | MR-Rv13 | acagtctcatcgcctgatcc | 399 | [2] |
|  | MR-1L/M 3' UTR | MR-Fw14 | tgcaca agagcaagtgatgc | MR-Rv14 | cagggctacagtgagatgagg | 396 | [2] |
|  | MR-1L/M 3' UTR | MR-Fw15 | aagaggaaaggaggggtctc | MR-Rv15 | gccactgaggcagagagtg | 400 | [2] |
|  | MR-1L/M 3' UTR | MR-Fw16 | ctgagactctgccctcttcc | MR-Rv16 | cagacgggcctaagaacaag | 391 | [2] |
|  | MR-1L/M 3' UTR | MR-Fw17 | acatctggcctggttgtacc | MR-Rv17 | act cccgcgctctcacag | 359 | [2] |
|  | MR-1L/M 3' UTR | MR-Fw18 | cagtttgccagccatattcc | MR-Rv18 | gaggctgtggctagagaagg | 390 | [2] |
|  | MR-1L/M 3' UTR | MR-Fw19 | agtcagccaggagccctcttt | MR-Rv19 | tgccacaagagaagcattgaca | 392 | [2] |
|  | MR-1L/M 3' UTR | MR-Fw20 | tcctcctgcctt gtctgc | MR-Rv20 | cctggaactgagatgggaaa | 388 | [2] |
| SLC2A1 (GLUT1) | exon 1 | sg382 | gtcctgcccacacacccctgac | sg380 | actcaccttgctgctgggctcc | 445 | this study |
|  | exon 2 | sg384 | gccagcccagtatcttgaagtcag | sg386 | gagactgtgggcatgtgtgatg | 366 | this study |
|  | exon 3-4 | sg388 | agagggacttggctcaagaacatgc | sg389 | gctgtgttctctggacctgtgtacc | 880 | this study |
|  |  | sg390 | tcagtgcggtcagggagtctgtac | sg391 | cagagaaggagccaatcatgcc | 951 | this study |
|  | exon 5-8 | sg392 | tcccatgtgaccgatgaggaaac | sg393 | tccctcactctccagaacctagc | 1309 | this study |
|  | exon 9 | sg396 | cacaacaaatccaggaccatcc | sg397 | atgcgtgcgggtgagtatagag | 377 | this study |
|  |  | sg396 | cacaacaaatccaggaccatcc | sg398 | ggagttgaggtcagcattcttgg | 493 | this study |
|  | exon 10 | sg400 | atcatgctgaggaggaaactgaggg | sg401 | cattgctggctggagaaaggag | 615 | this study |
|  |  | sg403 | GTGTGTCTTTCAGCAACTGTGTGG | sg402 | TGGGTAGGAAGAGATGGGAAGG | 752 | this study |

**References:**

1. Chen WJ, Lin Y, Xiong ZQ et al. (2011) Exome sequencing identifies truncating mutations in PRRT2 that cause paroxysmal kinesigenic dyskinesia. Nat Genet 43:1252-1255

2. Lee HY, Huang Y, Bruneau N et al. (2012) Mutations in the gene PRRT2 cause paroxysmal kinesigenic dyskinesia with infantile convulsions. Cell Reports
